# A GAN-BO-XGBoost model for high-quality patents identification

**DOI:** 10.1038/s41598-024-60173-9

**Published:** 2024-04-26

**Authors:** Zengyuan Wu, Jiali Zhao, Ying Li, Zelin Wang, Bin He, Liang Chen

**Affiliations:** 1 College of Economics and Management, China Jiliang University, No. 258, Xueyuan Street, Hangzhou, 310018 Zhejiang People’s Republic of China; 2 Syney Elevator (Hangzhou) Co., Ltd, No. 31, Yangcheng Street, Hangzhou, 310000 Zhejiang People’s Republic of China; 3 College of Optics and Electronic Technology, China Jiliang University, No. 258, Xueyuan Street, Hangzhou, 310018 Zhejiang People’s Republic of China

**Keywords:** High-quality patent identification, Imbalanced classification, Generative adversarial networks (GAN), Ensemble learning, Techniques and instrumentation, Information technology, Computer science, Mathematics and computing

## Abstract

The number of patents increases quickly, while more and more low-quality patents are emerging. It’s important to identify high-quality patents from massive data quickly and accurately for organizational R&D decision-making and patent layout. However, due to low percentage of high-quality patents, it is challenging to identify them efficiently. In order to solve above problem, we reconstruct the existing index system for identifying high-quality patents by adding 4 features from technological strength of patentees. Furthermore, we propose an improved model by integrating resampling technique and ensemble learning algorithm. First, generative adversarial networks (GAN) are used to expand minority samples. Second, Extreme Gradient Boosting algorithm (XGBoost) with Bayesian optimization (BO) is used to identify high-quality patents. For clarity, this model is called a GAN-BO-XGBoost model. To test the effectiveness of above model, we use patent data in field of lithography technology. Tenfold cross-validation is carried out to evaluate the performance between our proposed model and other models. The results show that GAN-BO-XGBoost model performs better and it’s more stable than other models.

## Introduction

In the knowledge economy era, the dominant role of technological innovation in social development is increasingly prominent. Intellectual property rights, serving as a tangible manifestation of technological breakthroughs, have evolved into a pivotal asset for nations worldwide, driving the enhancement of their international competitiveness and the cultivation of strategic resources. These rights play a crucial part in fostering economic and social progress, as well as advancements in science, technology, and culture. Patents, as an important component of intellectual property rights, serve as carriers of cutting-edge technologies and important indicators reflecting a country's scientific and technological strength and competitiveness. Therefore, guided by strategic direction and policy incentives in various countries, the international patent application volume has been increasing year by year. According to a report released by the World Intellectual Property Organization (WIPO), the total number of global Patent Cooperation Treaty (PCT) applications reached 278,100 in 2022, setting a record for the highest annual application volume.

However, behind the surge in patent applications, the number of low-quality patents such as "junk patents" and "short-lived patents" is also on the rise. The patent application process is not only time-consuming but also entails substantial costs. Despite numerous inventions securing patent protection, the conversion rate of these patents remains low, posing challenges for companies in realizing anticipated economic benefits and resulting in financial setbacks. Therefore, it is of great significance to identify high-quality patents from a large number of patents, understand technological trends, and track promising technologies. This can guide the research and development efforts of companies, promote the commercialization of patent achievements, and establish an optimal patent strategy and layout.

Scholars have delved into extensive research to address the challenge of identifying high-quality patents. Some scholars have constructed indicator systems by exploring patent quality indicators and determined indicator weights using evaluation methods. The filtration of high-quality patents is then based on the calculated weights^1^. However, these methods heavily rely on subjective expert judgment and demand a substantial investment of time and human resources. Therefore, machine learning technique is used to identify high-quality patents by more scholars. Wu et al.^2^ constructed an adaptive organization-self-organizing map-kernel principal component analysis-support vector machine (SOM-KPCA-SVM) model. This model can classify the quality of patents in the field of solar cells automatically. Trappey et al.^3^ selected indicators such as the number of IPCs (international patent classifications), the number of inventors, the number of claims, the number of patent citations, and the length of the patent text based on a literature review. They used deep neural networks to establish a classification model for patent quality and applied it to the field of technology mining in the manufacturing industry Internet of Things. Although existing studies have contributed to the identification of high-quality patents, they have overlooked the imbalanced data classification in patent datasets, where the number of high-quality patents is relatively lower compared to the number of low-quality patents. The majority of machine learning algorithms are designed based on balanced datasets. If imbalanced datasets are directly used to train the model, the classification results may be biased towards the majority class, leading to model distortion and a decrease in classification performance^4^.

Considering these gaps, our objective is to enhance the identification of high-quality patents by tackling the imbalanced data classification within patent data. Firstly, GAN is used to keep the class balance by generating the minority class samples, which help to solve the problem due to imbalanced data of high-quality patents. Then, the weight updating mechanism in ensemble learning is combined to identify high-quality patents. The BO is employed to find the optimal parameter combination for XGBoost. Finally, the Gan-BO-XGBoost model is proposed. Its performance is compared with other machine learning algorithms by Accuracy, Precision, Recall, F1-score, and AUC.

The rest is organized as follows in this article. In Sect. "Literature review", we review the related literature on the definition and identification of high-quality patent. In Sect. "Methodology", we propose the index system and our combined model. In Sect. "Empirical analysis", we implement the GAN-BO-XGBoost model to identify high-quality patents in field of lithography technology. In the last section, the conclusions are drawn, and ideas for future research are proposed.

## Literature review

### The definition of high-quality patent

At present, a universally accepted definition of "high-quality patents" is lacking. Some scholars have defined high-quality patents based on the interests of the country, companies, or individuals, considering the technological importance, legal stability, and market applicability of patents.

Thomas^5^ defined high-quality patents as those that are legally enforceable, can withstand challenges in patent litigation, and bring economic benefits as a tool for technology transfer. Allison & Hunter^6^ stated that high-quality patents not only had substantial innovation compared to existing technologies but also maintained their effectiveness when facing litigation. Hall & Harhoff^7^ believed that high-quality patents were not only protected by patent law to ensure the smooth implementation of the patented technology but also could be commercialized to generate economic benefits in practical applications. Tsao et al.^8^ had a similar view. They pointed out that high-quality patents had a high probability of facing patent litigation in the future, and an important feature for measuring patent quality was whether the patent faced and successfully passed litigation. Additionally, the pledging and investment of patent rights could also reflect their implementation capability and technological and economic contributions. Wei et al.^9^ argued that patent quality should be measured by its technological competitiveness and market potential. Therefore, high-quality patents not only could be used to improve the value of their innovation but also help organizations to win patent infringement litigation against competitors. Considering the relationship between products and patents, Guerrini^10^ suggested that patent quality should be defined from 3 aspects, including legal effectiveness, social utility and Commercial success.

### The identification of high-quality patent

In field of high-quality patent identification research, researchers choose indicators based on patent documents and information obtained during the patent application process, and construct an index system for identifying high-quality patents. Subsequently, evaluation methods are employed to determine the weights of each indicator, enabling the identification of high-quality patents. In the study conducted by Chiu & Chen^11^, indicators were selected from four dimensions, including technological features, technology market, cost, and product market. Then, the analytic hierarchy process (AHP) was used to evaluate patent quality. Baron & Delcamp^12^ established a patent quality evaluation system based on indicators, including the number of claims, the number of family patents, the authority of patent applicants, and the time of patent granting. They employed factor analysis to calculate the weights of each indicator and measured the quality of each patent using this evaluation method. Wang & Hsieh^13^ selected 40 indicators according to the literatures, then used factor analysis to identify 10 independent indicators. Furthermore, they determined the weights of each indicator using the Analytic Hierarchy Process (AHP). Finally, they classified the value of patents using fuzzy evaluation. Similarly, Leng & Zhai^14^ constructed patent quality evaluation indicators, obtained the weights of each indicator using the Analytic Hierarchy Process (AHP), and then evaluated the quality of medical patents using fuzzy comprehensive evaluation method.

In existing literatures, some scholars had developed a patent quality evaluation system through literature analysis and determined indicator weights for assessing patent quality. However, this method heavily relied on expert judgment, introducing a subjective element into the process. Moreover, a significant amount of time and human resources were necessary. Meanwhile, with the development of science and technology, some scholars tried to introduce more efficient and objective machine learning methods into patent quality evaluation. Choi et al.^15^ employed the construction of a feed-forward neural network model to predict the maximum possible economic lifespan of patents, thereby screening patents with high commercial potential. However, a single classification algorithm has limitations. For example, decision trees (DT) are less effective while predicting fields with continuity. Support Vector Machines (SVM) heavily rely on the performance of the kernel function. In contrast, ensemble learning cascading multiple weak learners to create a robust learner had better classification performance^16^. Kwon & Geum^17^ selected 17 indicators as input variables and used patent citation count as the output variable. They compared the performance between XGBoost and six machine learning algorithms. The results showed that XGBoost had the best classification performance.

Although significant progress has been made in the field of high-quality patent identification, two research gaps persist. Firstly, in the existing research focused on constructing patent quality evaluation indicators, most scholars primarily tend to prioritize technological, economic, and legal indicators, while neglecting indicators related to the technological strength of patentees. Several existing studies showed that patent quality depended on the technical capabilities of patentees. That is, patents from continuously innovative companies were more likely to be valuable^18^. Secondly, despite the advantages of machine learning methods in identifying high-quality patents, such as cost-effectiveness and efficiency, dealing with imbalanced data remains a challenging task. In cases where there is a significant imbalanced data, prediction outcomes are prone to favoring the majority class samples. However, in the context of data class imbalance, accurately identifying minority class samples is the key focus of research.

## Methodology

**Figure 1:**
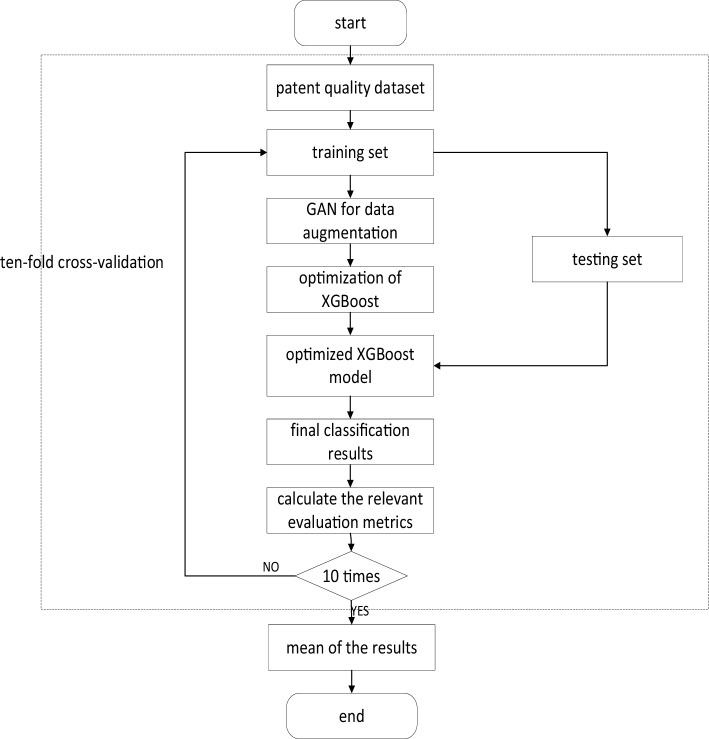
Flowchart of the high-quality patent identification model.

Given the complexity of identifying high-quality patents, the research method can be divided into three parts: data collection, selection of patent indicators, and construction of high-quality patent identification models. The detailed process is depicted in Fig. 1.

### Data collection

In this study, the Patsnap Global Patent Database was chosen as the source of patents which contains 170 million patent data from 158 countries and regions. We focused on patents in the field of photolithography technology. To ensure comprehensive coverage, relevant patents were extracted from this extensive database. Given the intricate complexities of photolithography technology, relying solely on a single IPC classification code or keyword search may result in omissions. Therefore, drawing on insights from relevant literature and expert interviews, we established corresponding keyword search expressions, complemented by IPC classification code searches.

### Selection and description of patent indicators

#### Output indicators

Due to the abstract nature of patent quality, it is difficult to measure patent quality directly. Therefore, it is necessary to select proxy indicators that can indirectly measure patent quality. The number of patent citations is often used to evaluate patent quality, based on the fundamental assumption that patent citations can be used to evaluate the competitiveness and impact of technological innovation^19^. By analyzing patent citations, it is possible to identify important patents. The more a particular patent is cited, the more important it is for innovative development, the higher its quality is perceived to be^20^. However, the relationship between patent quality and innovativeness is complex. There are some limitations and biases when patent citations are used to measure patent quality, as they fail to reflect the technological complexity inherent in patents and they cannot indicate potential factors^21^. Atallah & Rodriguez^22^ proposed that both the number of direct citations and the number of indirect citations should be taken into account in patent research. Using patents in the field of hard disk technology as samples, Wang et al.^23^ constructed a patent citation network and confirmed the close relationship between the centrality position of patents in the citation network and patent quality. Meanwhile, to overcome the limitations of a single direct citation network, Yang et al.^24^ restructured four types of citation relationships, including direct citation, indirect citation, coupling, and co-citation. In order to facilitate the selection of high-quality patents, a comprehensive citation network was constructed and applied in empirical research. The empirical results show that this approach is more comprehensive and accurate compared to relying solely on direct citation networks. Therefore, a patent citation network based on the citation relationships among patents was constructed in this study, and the closeness centrality in the patent citation network was utilized to measure patent quality.

#### Input indicators

According to the literature review, there were several indicators in patents that can affect their quality. In this study, input variables were categorized into four distinct dimensions, including Technological indicators, Economic indicators, Legal indicators, and the Technological strength of patentees. This classification provided a comprehensive framework for analyzing the various factors that influence patent quality. From these four dimensions, a total of 14 indicators were further explored and selected as input variables for machine learning. The final selected indicators were presented in Table 1.

**Table 1:** Input indicators for high-quality patent identification.

Primary indicators	Secondary indicators	Meaning or calculation method
Technological characteristics	Patent citation count	The number of prior patents cited by the target paten
	Literature citation count	The number of prior scientific literature references cited by the target patent
	Technical coverage scope	The number of IPC classification codes for the target patent
	The number of pages in the specification	The total number of pages in the target patent specification
	The number of inventors	The number of inventors of the target patent
Economic characteristics	The size of patent family	The number of patents filed for the target patent in different countries or regions
	Whether the patent has been transferred	Whether there has been a transfer of ownership for the target patent
Legal characteristics	Application duration	The difference between the target patent's grant date and application date
	The number of claims	The number of claims in the target patent
	The number of litigations	The number of litigations involving the target patent
Technological strength of patentees	The number of patent owners	The number of patent owners of the target patent
	Overall activity level	The total number of patents issued by the patent owner
	Core activity level	The total number of patents issued by the patent owner in the respective field
	Core technological influence	The total number of citations received by the patents issued by the patent owner in the respective field

##### Technological indicators

Patent citation count refers to the number of times a patent is cited by other patents, which reflects the technological novelty of the patent. If a patent cites other granted patents, it indicates that the patent has introduced specific improvements and enhancements compared to the cited patents at a technological level, indicating a higher level of technological quality^25^.

Literature citation count refers to the number of scientific literature citations received by a patent, including scientific papers, conference proceedings, and books. A higher literature citation count indicates a greater reliance of the patent on scientific literature achievements and suggests a higher quality of the patent. Therefore, the literature citation count can reflect the patent quality and is positively correlated with the patent^26^.

Technological coverage scope refers to the range of technology covered by a specific patent. The IPC classification codes are internationally recognized as a relatively objective indicator of technology cross-domain information^27^. Therefore, we use the quantity of 4-digit IPC classification codes to represent the size of technological coverage scope. Furthermore, the results of relevant research have shown a strong positive correlation between the number of IPC categories and the technological and economic value of patents^28^.

The specification is used to describe the structure, technical features, and usage methods of a particular patent technology. The number of pages in the specification refers to the total number of pages in the specification section of the patent document. A greater number of pages in the specification indicate that the technology covered by the patent is more complex and poses a higher level of difficulty for imitation.

The number of inventors reflects the collaborative nature of a patent. The development of a patent often involves multiple disciplines, requiring the collision of ideas from researchers with different knowledge backgrounds. Therefore, an increased number of inventors indicates a higher level of complexity in the technology, a greater demand for diverse knowledge, and a higher quality of the patent^29^.

##### Economic indicators

If inventors wish to protect their invention in multiple countries or regions, they must file corresponding patent applications in each country or region^15^. The number of patents filed for the same invention in multiple countries or regions is referred to as the patent family size. A larger patent family size indicates a stronger ability to strategically position patents, which can provide a competitive advantage in the market and lead to higher economic benefits^30^.

Patent transfer refers to the act of transferring the ownership or rights of a patent from the patent holder to the assignee. As an important form of commercializing technological achievements, patent assignment can reflect the economic quality of a patent. The existence of patent assignments indicates that the patent holds certain market economic value^31^.

##### Legal indicators

Application duration refers to the duration from the filing date to the patent grant date. A long application timeline indicates that the patent applicant has devoted significant effort to inventing their patent, and the examiner needs to conduct a thorough examination. As a result, the corresponding patent tends to be more stable^32^.

The number of claims refers to the quantity of claims in a patent, including independent claims and dependent claims, which indicates the legal scope of protection conferred by the patent^33^. Fischer and Leidinger found that patents with a larger number of claims are more likely to face invalidation proceedings^34^. Furthermore, some studies suggested that the number of claims could reflect the value or quality of a patent. Lee et al.^35^ confirmed a strong positive correlation between the quantity of claims and patent quality using the "zero-inflation model."

The number of patent litigations reflects the legal effectiveness of a patent. Patents with higher technical content and stronger novelty are more likely to face infringement issues^36^.

##### Technological strength of patentees

After patent grant, the ownership of the patent is shared among multiple patent holders. The number of patentees indirectly reflects the scale of investment from the patent development organization. A greater number of patent holders can offer increased support and protection in the event of patent litigation.

Overall activity level refers to the total number of granted patents that a patent holder has applied for. It reflects the patent holder's technological innovation capability. A higher value of the overall technology indicator indicates that the patent holder has a faster pace of technological advancement and higher patent quality.

Core activity level refers to the total number of granted patents that a patent holder has applied for in a specific field. This metric serves as an indicator of the patent holder's knowledge level within that particular domain. A higher core technology value indicates that the patent holder has a deeper understanding and mastery of core knowledge in that field, leading to higher patent quality.

The total number of citations received by patents granted to a patent holder in a specific field reflects the company's technological strength or competitive position in that domain. The greater the impact of a patent holder's core technology is, the higher the patent quality is.

### High-quality patent identification model

Based on the high-quality patent identification indicator system, we propose a high-quality patent identification model to rapidly and accurately identify high-quality patents. The model consists of two main steps: augmentation of data sample based on GAN and the employment of the XGBoost algorithm with BO.

#### Input indicators

**Figure 2:**
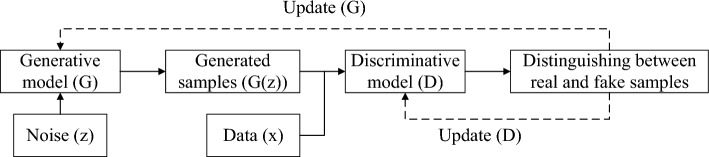
The training process of GAN.

Generative Adversarial Networks (GAN) is a generative model proposed by Goodfellow et al. in 2014 based on game theory^37^. In imbalanced datasets, GAN-based oversampling technique is used to learn well the global distribution of minority class samples and generate highly realistic and diverse minority class samples, helping to reduce redundant and duplicate samples in the generated samples and make up for the shortcomings of traditional oversampling technique^38^. The GAN model consists of a generator (G) and a discriminator (D). The generator generates new samples from random noise inputs, while the discriminator determines whether the input is a real sample or a fake sample generated by the generator. As shown in Fig. 2, the training process of GAN involves a game between G and D. The objective of G is to generate samples that resemble real samples, while the objective of D is to accurately distinguish between real and generated samples. To win the game, the generator continuously improves its generation capability, and the discriminator enhances its discrimination ability. Eventually, an equilibrium state is reached, where G can generate counterfeit samples that closely resemble real samples. The objective function of GAN is defined as follows:

1 $$\underset{G}{{\text{min}}}\;\underset{D}{{\text{max}}}F\left(D,G\right)={E}_{x\sim {p}_{m}}\left[{\text{ln}}\left(D\left(x\right)\right)\right]+{E}_{z\sim {p}_{z}}[{\text{ln}}\left(1-\left({\text{G}}\left({\text{z}}\right)\right)\right).$$

Among them,$F(D,G)$ represents the loss function, ${P}_{m}$ denotes the distribution of real samples. ${P}_{z}$ represents the distribution of generated samples. $G(z)$ denotes the counterfeit samples generated by from input noise $z$. $D(x)$ represents the probability that classifies $x$ as a real sample. The goal of $G$ is to minimize $F(D,G)$ as much as possible, while the goal of $D$ is to maximize $F(D,G)$ as much as possible. These two objectives alternate during training, ultimately leading to an optimal solution for the generative model.

#### XGBoost algorithm with Bayesian optimization

##### Principles and applicability analysis of XGBoost algorithm

Extreme gradient boosting (XGBoost) stands out as a prominent ensemble algorithm within the Boosting family. It works by adding new decision trees to fit the residuals of the previous predictions, and then accumulates the predictions of all trees to obtain the final value of the predictive model. In recent years, XGBoost has been widely applied in the field of data mining due to its high training speed and prediction performance. The algorithm was developed by Chen & Guestrin^39^ as an optimization of Gradient Boosting Decision Trees (GBDT). The improvement lies in the addition of a regularization term to the loss function, which effectively prevents overfitting and enhances generalization capability. Additionally, XGBoost employs a second-order Taylor expansion of the objective function, making the approximate optimization of the objective function closer to the actual values and thus improving prediction performance. The principles of the XGBoost algorithm are as follows:

Setting the objective function as follows:

2 $$Obj={\sum }_{i=1}^{n}L({y}_{i},{\widehat{y}}_{i}^{(t)})+{\sum }_{i=1}^{t}\Omega \left({f}_{t}\right).$$

Among them, ${\widehat{y}}_{i}$ represents the predicted value of the ith sample, and ${y}_{i}$ represents the actual value of the ith sample. $L({y}_{i},{\widehat{y}}_{i}^{\left(t\right)})$ represents the loss function, which is the difference between the predicted value and the actual value. ${\sum }_{i=1}^{t}\Omega ({f}_{t})$ represents the regularization term, and its calculation process is as follows:

3 $$\Omega \left({f}_{t}\right)=\gamma T+\frac{1}{2}\lambda {\sum }_{j=1}^{T}{\omega }_{j}^{2}.$$

Among them, $\lambda$ represents the penalty coefficient. $T$ represents the number of leaf nodes in a tree. $\omega$ represents the score obtained by a leaf node. $\lambda$ and $\gamma$ are the weighting coefficient used to prevent model overfitting.

In the tth iteration, the predicted value for the ith sample is represented as follows:

4 $${\widehat{y}}_{i}^{(t)}={\widehat{y}}_{i}^{(t-1)}+{f}_{t}\left({x}_{i}\right).$$

${f}_{t}({x}_{i})$ represents the newly added base classifier in the iteration, so the objective function is rewritten as follows:

5 $$Obj={\sum }_{i=1}^{n}L({y}_{i},{\widehat{y}}_{i}^{\left(t-1\right)}+{f}_{t}\left({x}_{i}\right)+\Omega \left({f}_{t}\right).$$

Expanding the objective function with a second-order Taylor series approximation, and using ${g}_{i}$ to represent the first-order derivative of the loss function, and ${h}_{i}$ to represent the second-order derivative term of the loss function, the optimization is obtained by removing the constant term.

6 $$Obj\cong {\sum }_{i=1}^{n}[L({y}_{i},{\widehat{y}}_{i}^{\left(t-1\right)})+{g}_{i}{f}_{i}({x}_{i})+\frac{1}{2}{h}_{i}{f}_{t}^{2}({x}_{i})]+\Omega ({f}_{t}).$$

Let ${G}_{j}=\sum_{i\in {I}_{j}}{g}_{i}$ and ${H}_{j}=\sum_{i\in {I}_{j}}{h}_{i}$ represent the sum of the first and second partial derivatives of the leaf node predictions. The objective function can be updated as follows:

7 $$Obj={\sum }_{j=1}^{T}\left[{G}_{j}{w}_{j}+\frac{1}{2}\left({H}_{j}+\lambda \right){w}_{j}^{2}\right]+{\lambda }^{T}.$$

Setting the derivative to 0 results in the optimal solution for ${w}_{j}$. Finally, substituting the optimal solution into the objective function, we get:

8 $$Obj=-\frac{1}{2}{\sum }_{j=1}^{T}\frac{{G}_{j}^{2}}{{H}_{j}+\lambda }+{\gamma }^{T}.$$

XGBoost is an ensemble learning algorithm based on improved gradient boosting decision trees. It improves the overall classification performance by integrating the classification results of multiple decision trees. Its main characteristics are as follows:

Firstly, it exhibits excellent classification performance. The XGBoost algorithm uses a second-order Taylor expansion to unfold the loss function, which effectively makes the approximate optimization of the objective function closer to the actual values and enhances the model's ability to identify high-quality patents.

Secondly, it has a fast execution speed. After each iteration of the XGBoost algorithm, the weights of the leaf nodes are multiplied by a coefficient. The step aims to mitigate the influence of each individual tree, allowing for greater flexibility and space for subsequent learning. Furthermore, an approximate histogram algorithm is employed during tree node splitting to efficiently generate candidate split points.

Lastly, it can solve the problem of linear correlation among independent variables. According to the previous analysis, it is evident that various factors influence patent quality, and intricate relationships may exist among them. For example, there may exist potential relationships among the number of patent owners, overall activity level, and core activity level. The correlation among these factors can lead to unstable parameter estimation in the model. The XGBoost algorithm addresses this issue by introducing the idea of regularization. It adds a penalty term to the objective optimization equation of parameter estimation to control the complexity of the model, thereby reducing model overfitting.

##### The XGBoost algorithm based on Bayesian optimization

**Figure 3:**
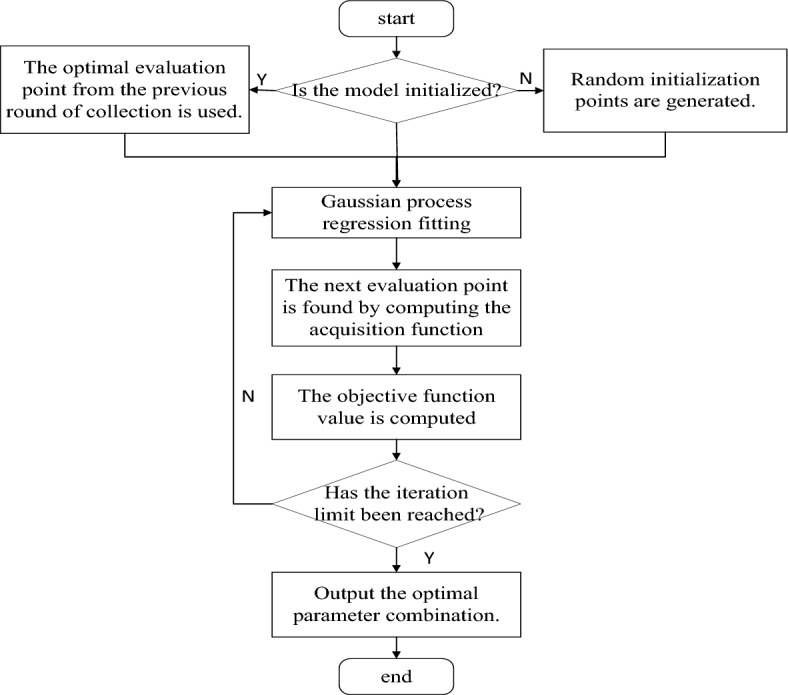
Bayesian optimization workflow diagram.

XGBoost, as an ensemble algorithm, exhibits superior classification performance. However, the performance of this algorithm is influenced by key parameters. In practical operations, it requires continuously adjusting the parameter values to obtain the optimal parameter combination. Manual parameter tuning in the past had certain subjectivity and was prone to losing the optimal parameter combination, which reduce the classification performance of the model. Therefore, in this study, we utilized the Bayesian optimization algorithm to find the optimal parameter combination for XGBoost. The process flowchart for Bayesian optimization is depicted in Fig. 3. The core idea of this method is to leverage the rapid optimization capability of the Bayesian optimization algorithm. The AUC value of the model serves as the output of the objective function. Drawing from the previous search results, the parameters of the XGBoost model are continuously optimized to obtain the best parameter combination, thereby improving the classification performance of XGBoost. The steps of the BO-XGBoost method are as follows:

- Set the range of XGBoost parameters to be optimized and the number of iterations for Bayesian optimization.
- Check if the model is initialized. If it is not initialized, randomly generate a set of parameters. If it is already initialized, use the points collected from the previous round.
- Select the point with the highest probability using the acquisition function and calculate the corresponding true value for that point.
- When the iteration limit is not reached, update the Gaussian model. Select the next point with the highest probability and input it into the Gaussian model. Iterate this process until the parameter combination that optimizes the objective function is found.

## Empirical analysis

### Data source and preprocessing

Chips belong to the national high-precision technology industry and are a strategic battleground for countries worldwide. Simultaneously, chip products are involved in various aspects of our lives. Photolithography technology refers to the process of transferring patterns from a mask to a wafer using photoresist under the influence of light. It is the most complex and critical process in chip manufacturing. The development of photolithography technology can improve the computational speed and storage capacity of chips. It is the key technology for driving the development of the chip industry. Therefore, to break through the technological blockade, the development of photolithography technology is very important.

In this study, we utilized the PatSnap patent database to obtain patent data in the field of photolithography. Considering the complexity of photolithography technology, a sole reliance on IPC classification codes or keywords for retrieval may lead to some omissions. Therefore, based on the review of relevant literature and expert recommendations in the industry, a combined approach using IPC classification codes and keywords was employed to retrieve relevant patents. The final patent retrieval strategy was determined as follows (TA:(lithography OR microlithograph OR photolithography OR stepper OR scanner OR step—AND—scan OR step—AND—repeat) AND (lens OR mask OR photomask OR resist OR photoresist OR euv OR duv OR extreme ultraviolet) IPC:(G02B OR G03B OR G03F OR H01L)). The experimental data consisted of patent data granted from 2011 to 2021, yielding a total of 11,671 patent records, among which 1167 patents were classified as high-quality patents, and 10,504 patents were classified as low-quality patents.

In order to identify high-quality patents, it is necessary to categorize patent quality in training set. Based on the combination of direct citation relationships and indirect citation relationships, a patent citation network is constructed by considering the citing relationships among patents. The closeness centrality of each patent is calculated to represent its quality. After calculating the closeness centrality for all patents, the top 10% of patents with the highest closeness centrality are defined as high-quality patents. If the closeness centrality value exceeds the top 10%, the output variable for that patent is set to 1. Otherwise, it is set to 0.

### Metrics of model evaluation

To draw reliable and robust conclusions about the predictive performance of the proposed method, three performance metrics are used: accuracy, precision, recall, F1-score, and area under curve (AUC) of the ROC curve.

A confusion matrix is a specific matrix used to assess the performance of a model. The structure of the confusion matrix is described in Table 2.

**Table 2:** Confusion matrix.

Actual class	Predicted results	
	Predicted positive	Predicted negative
Actual positive	TP	FN
Actual negative	FP	TN

Positive samples represent high-quality patents, while negative samples represent low-quality patents. Based on the confusion matrix, the above four metrics can be computed.

#### Accuracy

Accuracy indicates that the ratio of correctly classified samples to the total number of samples and it is one of the most straightforward evaluation metrics in classification models. The greater the value of Accuracy, the better the model’s predictive performance.

9 $$Accuracy=\frac{TP+TN}{TP+TN+FP+FN}.$$

#### Precision

Precision refers to the ratio of the number of correctly predicted positive samples to the number of all predicted positive samples. In other words, precision measures how many of the predicted positive examples are actually true positives.

10 $$Precision=\frac{TP}{TP+FP}.$$

#### Recall

Recall, also known as sensitivity or true positive rate, indicate that the proportion of actual positives that are correctly predicted as positive. A higher recall indicates fewer misclassified samples from the minority class. The formula is as follows:

11 $$Recall=\frac{TP}{TP+FN}.$$

#### F1-score

The F1 score is a metric that calculates the weighted harmonic mean of recall and precision. It will only be high if both recall and precision values are relatively large. Therefore, the F1 score comprehensively reflects the classification performance of the algorithm for both positive and negative samples.

12 $$F1-score=\frac{2\times Prescision\times Recall}{Precision+Recall}.$$

#### AUC

AUC refers to the area under the ROC curve, which is a plot of the true positive rate (TPR) against the false positive rate (FPR). The ROC curve depicts the variation of the FPR and TPR under different parameter settings. A curve that is closer to the top-left corner indicates better classifier performance. The AUC ranges from 0 to 1. A higher value indicates better classifier performance.

13 $$TPR=\frac{TP}{TP+FN},$$

14 $$FPR=\frac{FP}{FP+TN}.$$

### Experimental results

#### Model parameter optimization

Although the XGBoost ensemble algorithm can improve classification performance by cascading multiple weak classifiers, it has a notable drawback in practical applications. The performance of XGBoost is closely related to the settings of its parameters, and traditional manual tuning is time-consuming and prone to overlooking the optimal solution. Consequently, the Bayesian optimization method is employed to search for the optimal parameter combination for the XGBoost model. The key parameter intervals for Bayesian optimization are set as shown in Table 3.

**Table 3:** XGBoost parameter optimization.

Parameter	Meaning	Optimization space
n_estimators	The number of decision trees	[10, 250]
learning_rate	Learning rate	[0.01, 0.5]
max_depth	Maximum depth of trees	[1, 14]
gamma	Minimum loss reduction	[0, 1]

**Figure 4:**
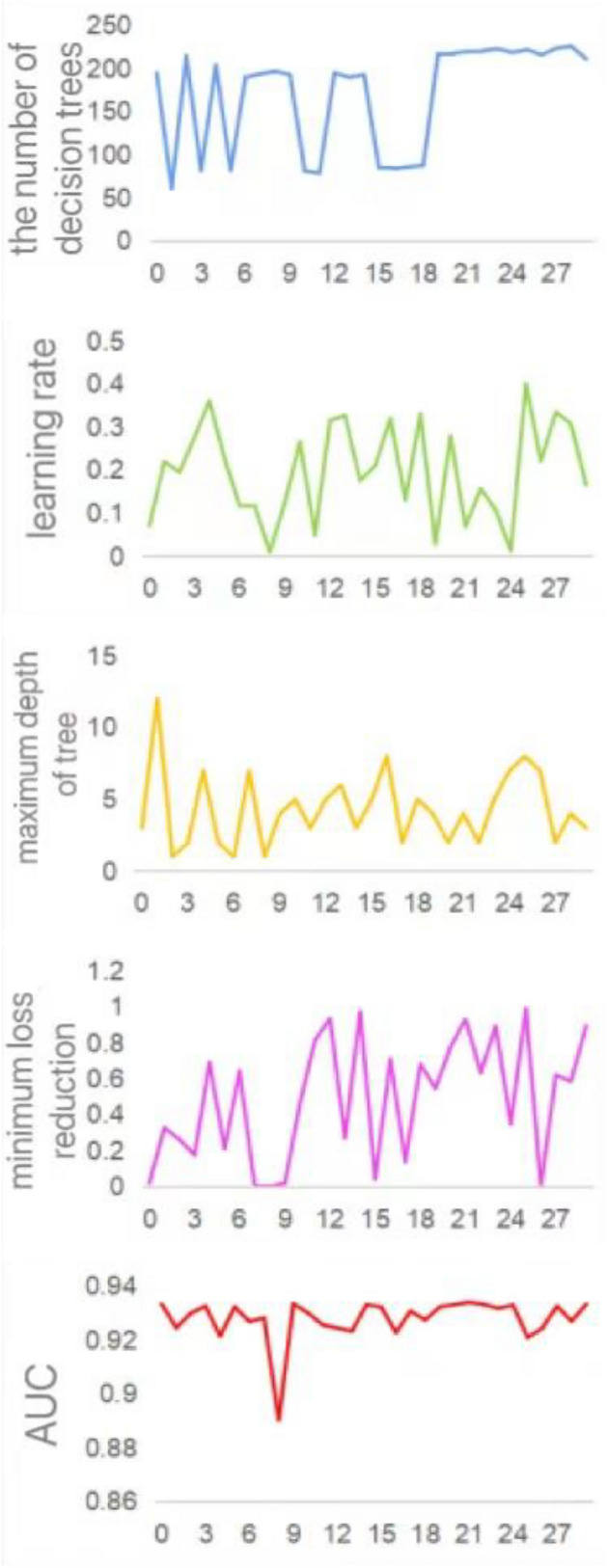
Iteration process of parameters.

Once the parameter optimization intervals are determined, the dataset is divided into training and testing sets. The training set is used to train the model, with the AUC value on the testing set serving as the objective function. The maximum number of iterations is set to 30 to initiate the parameter optimization process. The iterative process of each parameter is shown in Fig. 4, where x-axis represents the iteration number, and the y-axis represents the key parameters and their value ranges. From the graph, it can be observed that at the 21st iteration, the model achieves the highest AUC value of 0.934. At this point, the corresponding number of decision trees is 220, the learning rate is 0.069, the maximum depth of trees is 4, and the minimum loss reduction is 0.930. This parameter combination represents the optimal parameter combination required.

After determining the optimal parameter combination, the values of the optimal parameters are input into the XGBoost model, resulting in the BO-XGBoost model. The data sampled by GAN is inputted into XGBoost model and BO-XGBoost model for training. Then, ten-fold cross-validation is employed, and the performance is measured by taking the average of ten experiments. The comparison of model performance before and after parameter optimization is shown in Table 4.

**Table 4:** The analysis of model performance.

Model	Accuracy	Precision	Recall	F1-score	AUC
GAN-XGBoost	0.798	0.758	0.867	0.808	0.921
GAN-BO-XGBoost	0.866	0.814	0.879	0.846	0.935

From the Table 4, it can be observed that compared to before parameter tuning, the XGBoost model has shown significant improvements in all metrics after parameter optimization, enhancing the effectiveness of identifying high-quality patents. After parameter optimization, the accuracy of the model has increased by 6.8%, reaching 0.866. This increase indicates that the model possesses robust discriminative power and is capable of efficiently identifying high-quality patents. In addition, the precision of the model has improved by 5.6%, reaching 0.814, and the recall rate has increased by 1.2%, arriving at 0.879. The high precision and recall rate indicate that the model can identify a greater number of high-quality patents. The F1-score metric has improved by 3.8%, reaching 0.846. This indicates that the model does not overly prioritize high-quality patents at the expense of ignoring low-quality patents, thus maintaining a good balance in classifying both types. Furthermore, we use AUC to measure the model's ability to identify patent quality. It can be observed that the parameter-optimized model achieves a high AUC of 0.935, which is a 1.4% improvement compared to the original model. This means that our model can effectively identify high-quality patents, even while dealing with imbalanced datasets. It has high prediction rates and a minimal number of errors.

#### The analysis of model comparison

To further evaluate the effectiveness of the model, we compare it with other commonly used machine learning algorithms. The sampled patent dataset is divided into training and testing sets, following a 9:1 ratio. The same ten-fold cross-validation is performed, and the results are averaged over the ten testing sets for comparison analysis. The comparative results are shown in Table 5.

**Table 5:** The results of model comparison.

Model	Accuracy	Precision	Recall	F1-score	AUC
LR	0.772	0.552	0.057	0.103	0.521
DT	0.715	0.389	0.411	0.399	0.609
KNN	0.748	0.411	0.214	0.282	0.561
BP neural network	0.701	0.467	0.238	0.315	0.557
RF	0.785	0.574	0.257	0.355	0.600
GAN-BO-XGBoost	0.866	0.814	0.879	0.846	0.935

It can be observed from the Table 5 that the GAN-BO-XGBoost model achieves the highest accuracy, reaching 86.6%, while the values of all other models are below 80%. This indicates that the GAN-BO-XGBoost model can effectively distinguish high-quality patents within the overall sample. In addition, in terms of precision and recall, the GAN-BO-XGBoost model also demonstrates a significant advantage over other models, which is substantially higher. This indicates that the GAN-BO-XGBoost model possesses excellent capabilities to minimize the number of false negatives (FN) and false positives (FP), thereby identifying high-quality patents. This capability holds significant importance for businesses in practical scenarios, enabling them to swiftly and precisely identify high-quality patents for targeted nurturing and development.

Regarding the F1-score, the other five models have relatively low scores, while GAN-BO-XGBoost performs best with a score of 84.6%. This indicates that our model not only identifies high-quality patents but also exhibits good classification performance for a large number of low-quality patents. In terms of the AUC metric, only GAN-BO-XGBoost achieves an AUC value above 90%, reaching 93.5%, while the other models show little variation and all fall below 65%.

Overall, our model demonstrates good performance in all metrics, which are commonly used to evaluate classification problems with imbalanced data. Based on the analysis, when compared to the mainstream methods currently used in the field of patent quality assessment, our model has proven to be efficient and effective.

#### The comparison of metric system

One of the research focuses in this paper is to introduce the indicator of patentee's technological strength, based on the three-dimensional indicator system of technological, economic, and legal characteristics. Therefore, the purpose of this section is to compare the metric systems and validate the effectiveness of incorporating the indicator of patentee's technological strength in enhancing the ability to identify high-quality patents.

The experiment compares the high-quality patent identification metric system constructed in this paper, where 4 indicators of the patentee’s technological strength are added, with the existing high-quality patent identification metric system without these indicators. In addition to the GAN-BO-XGBoost model proposed in this paper, other models such as logistic regression (LR), decision tree (DT), KNN, BP neural network, random forest, and XGBoost are selected to construct models. Accuracy, precision, recall, F1-score, and AUC are chosen as evaluation metrics. The ten-fold cross-validation method is used, and the results are measured by calculating the average across ten iterations. The comparative results are shown in Table 6.

**Table 6:** The comparison of metric system.

Model	Accuracy (%)			Precision (%)			Recall (%)			F1-score (%)			AUC (%)		
	N	Y	D	N	Y	D	N	Y	D	N	Y	D	N	Y	D
LR	77.0	77.2	0.2	50.0	55.2	5.2	4.2	5.7	1.5	7.8	10.3	2.5	51.2	52.1	0.9
DT	69.7	71.5	1.8	35.3	38.9	3.6	37.7	41.1	3.4	36.3	39.9	3.6	58.5	60.9	2.4
KNN	75.0	74.8	− 0.2	41.0	41.1	0.1	22.2	21.4	− 0.8	29.0	28.2	− 0.8	56.5	56.1	− 0.4
BP neural network	69.0	70.1	1.1	37.3	46.7	9.4	5.3	23.8	18.5	9.0	31.5	22.5	51.7	55.7	4.0
RF	76.7	78.5	1.8	48.9	57.4	8.5	19.6	25.7	6.1	27.8	35.5	7.7	56.6	60.0	3.4
XGBoost	77.8	78.1	0.3	49.0	50.2	1.2	15.3	19.3	4.0	23.9	29.5	5.6	75.7	78.3	2.6
GAN-BO-XGBoost	83.2	86.6	3.4	80.8	81.4	0.6	82.8	87.9	5.1	81.6	84.6	3.0	91.9	93.5	1.6

“Y” indicates the model with patentee's technological strength, "N" indicates the model without patentee's technological strength, and "D" indicates D-value.

From Table 6, it can be observed that most of the differences are greater than 0, indicating that the classification performance has been improved after adding the indicators of patentee's technological strength. Although there are a few models that show a slight decrease in performance after introducing the indicator, the decrease is minimal. Specifically, in terms of the accuracy metric, aside from the KNN model which exhibits a difference of − 0.2%, the means of all other models have shown improvement, indicating an enhancement in the capability of the models to identify high-quality patents. Regarding the precision and recall metrics, except for a slight decrease in the recall rate of the KNN model, all other models demonstrate positive differences, suggesting a reduction in the likelihood of misclassification of high-quality patent samples. In terms of the F1-score, while the difference for the KNN model is − 0.8%, the differences for the remaining models are positive and all exceed 2%. Comparatively, the degree of decrease is relatively low, indicating that the overall performance of the models in classification tasks has improved. In terms of the AUC metric, only the KNN model has a negative difference, while the other models have positive differences. Therefore, the performance of the model can be improved by adding the indicators of patentee's technological strength.

## Discussion

The main contributions of this article are as follows. Firstly, the indicator system is optimized in this article. Based on reviewing existing research, 4 indicators of patentee's technological strength were added to reconstruct the existing index system for identifying high-quality patents. Secondly, in order to address the challenge of identifying high-quality patents caused by data imbalance, we introduce GAN to oversample. Furthermore, in order to overcome the bias of classification results, we introduce Bayesian algorithm to improve the classification performance of XGBoost. These will help to improve the model's ability to identify high-quality patents.

## Conclusion

High-quality patent identification is a key factor in enhancing the innovation capabilities of nations and enterprises. To address the research gaps in existing research on patent quality evaluation, we have introduced the indicators of the patentee's technological strength and developed a metric system for evaluating high-quality patent identification. Additionally, according to the imbalanced data in the categories of high-quality patent data, we propose the GAN-BO-XGBoost model. In this model, GAN is employed to augment samples of the minority class, reducing the imbalance in patent data. The Bayesian optimization algorithm (BO) is employed to find the optimal parameter combination for XGBoost.

The performance of GAN-BO-XGBoost model is tested by photolithography patent data from the Patsnap patent database. According to empirical results, several conclusions are drawn. Firstly, the high-quality patent identification indicator system with patentee's technological strength can identify high-quality patents better. The patentee’s technological strength has a significant impact on patent quality, as patentees with stronger technological capabilities are more likely to develop high-quality patents. Therefore, when evaluating patent quality, it is essential to add the indicators of patentee’s technological strength. Secondly, the GAN-BO-XGBoost model proposed in this study performs better in high-quality patent identification. Compared to LR, DT, KNN, BP neural networks, and random forest models, the model proposed in this study exhibits the best performance in terms of accuracy, precision, recall, F1-score, and AUC metrics. The Accuracy reaches 86.6%, the Precision reaches 81.4%, the Recall reaches 87.9%, the F1-score reaches 84.6%, and the AUC value reaches 93.5%. In practice, this model can be applied to identify high-quality patents.

The high-quality patent identification model proposed in this article also has certain limitations. When selecting indicators for high-quality patent identification, only the structured features of patents are considered. However, a lot of information related to patent quality is also contained in textual contents such as patent abstracts and titles, which should also be incorporated into the indicator system. Therefore, in future research, further investigation can be conducted in feature engineering by extracting patent text features using natural language processing methods. A more comprehensive and robust model with better performance of identifying high-quality patents can be proposed.

Additionally, we only test the performance of the GAN-BO-XGBoost model using the patent data in the field of "lithography" technology. The generalizability of this model in other technological domains should be tested. Collecting more patent samples from different domains can improve our model proposed in this study.

## Data Availability

All data generated or analyzed during this study are obtained from the corresponding author upon reasonable request.
